# Different responses of mice and rats hippocampus CA1 pyramidal neurons to *in vitro* and *in vivo*-like inputs

**DOI:** 10.3389/fncel.2023.1281932

**Published:** 2023-12-07

**Authors:** Paola Vitale, Fabio Librizzi, Andrea C. Vaiana, Elisa Capuana, Maurizio Pezzoli, Ying Shi, Armando Romani, Michele Migliore, Rosanna Migliore

**Affiliations:** ^1^Institute of Biophysics, National Research Council, Palermo, Italy; ^2^Laboratory of Neural Microcircuitry, École Polytechnique Fédérale de Lausanne (EPFL), Lausanne, Switzerland; ^3^Blue Brain Project, École Polytechnique Fédérale de Lausanne (EPFL), Campus Biotech, Geneva, Switzerland

**Keywords:** pyramidal neurons, hippocampus, rodent comparison, electrophysiological features, computational modeling

## Abstract

The fundamental role of any neuron within a network is to transform complex spatiotemporal synaptic input patterns into individual output spikes. These spikes, in turn, act as inputs for other neurons in the network. Neurons must execute this function across a diverse range of physiological conditions, often based on species-specific traits. Therefore, it is crucial to determine the extent to which findings can be extrapolated between species and, ultimately, to humans. In this study, we employed a multidisciplinary approach to pinpoint the factors accounting for the observed electrophysiological differences between mice and rats, the two species most used in experimental and computational research. After analyzing the morphological properties of their hippocampal CA1 pyramidal cells, we conducted a statistical comparison of rat and mouse electrophysiological features in response to somatic current injections. This analysis aimed to uncover the parameters underlying these distinctions. Using a well-established computational workflow, we created ten distinct single-cell computational models of mouse CA1 pyramidal neurons, ready to be used in a full-scale hippocampal circuit. By comparing their responses to a variety of somatic and synaptic inputs with those of rat models, we generated experimentally testable hypotheses regarding species-specific differences in ion channel distribution, kinetics, and the electrophysiological mechanisms underlying their distinct responses to synaptic inputs during the behaviorally relevant Gamma and Sharp-Wave rhythms.

## 1 Introduction

A neuron’s function, activity, signal integration and propagation properties depend on specific morphological and electrophysiological characteristics. The ultimate goal of studying neuronal functions is to understand the inner workings of the human brain. However, given the obvious problems in obtaining detailed data from human neurons, especially under physiological conditions, the vast majority of experimental investigations at cellular level are performed on rodent cells. This raises the question of how and to what extent it is possible to apply these results not only to humans but also to other species. This is important, because it has been suggested (Miller et al., 2010; Uhl and Warner, 2015; Zeiss, 2017; Zhao and Bhattacharyya, 2018) that the use of animal cell models whose properties may not match those of humans, could lead to therapies and predictions that are not directly transferable to human cells. For this reason, the use of cells that are as similar as possible to human cells in terms of electrophysiological properties could enable faster and more efficient development of new insights and strategies to improve brain health.

Mice and rats are the preferred species for studying brain function but, for example, the morphological properties of their pyramidal cells are markedly different (Routh et al., 2009); this is not limited to scalar or geometric properties, but includes differences in topological complexity that can lead to species-dependent neuronal functions, as suggested by behavioral studies showing that mice have different and simpler strategies for learning spatial information and spatial navigation than rats (Whishaw, 1995; Frick et al., 2000). Human neurons, which require greater temporal precision and computational cost to efficiently perform their functions, exhibit an even greater morphological complexity (Lund et al., 1993; Malach, 1994; Elston et al., 1999; Elston, 2003; Goriounova et al., 2018). Electrophysiological recordings have also shown some significant differences between mice and rats, although they were not clearly characterized in terms of differences in expression and/or distribution of ion channels (Routh et al., 2009).

Here we present a detailed comparison of mouse and rat hippocampal CA1 pyramidal cells, based on electrophysiological data, morphologies, and computational models. This allowed a deeper understanding of the aspects that characterize their input/output properties, of how these may be the result of a different distribution of somatodendritic ion channels or synaptic inputs, and how and to what extent they can be compared with (or extrapolated to) human neurons. The results show significant physiological differences in the process of synaptic integration between mice and rats CA1 hippocampal pyramidal cells and suggest that rats should be preferentially used when extrapolating results to humans.

## 2 Materials and methods

### 2.1 Hippocampal slice preparation

All animal experimentation was conducted according to the Swiss National Institutional Guidelines on Animal Experimentation and approved by the Swiss Cantonal Veterinary Office Committee for Animal Experimentation (License number: VD3389).

Young (p13-p16), male mice C57BL/6J wild type were placed in deep anesthesia (isoflurane) and then sacrificed. The brain was quickly extracted and placed in cold oxygenated (O_2_ 95%; CO_2_ 5%) cutting extracellular solution (in mM: 213.0 sucrose, 2.5 KCl, 10.0 MgCl_2_, 1.25 NaH_2_PO_4_, 0.5 CaCl_2_, 25.0 glucose, 25.0 NaHCO_3_). In the same solution and with the assistance of a semiautomatic vibrating blade microtome (Leica VT1200S), acute coronal sections 300 μm thick were obtained. The slices then were placed at room temperature in oxygenated (O_2_ 95%; CO_2_ 5%) normal extracellular solution (in mM: 125.0 NaCl, 2.5 KCl, 1.0 MgCl_2_, 1.25 NaH_2_PO_4_, 2.0 CaCl_2_, 25.0 glucose, 25.0 NaHCO_3_) for at least 1 h to recover before recording. All chemicals were obtained from Sigma-Aldrich.

A comparison of differences with rat and human experimental protocols, used as reference, was reported in Supplementary Table 1.

### 2.2 Staining and reconstruction techniques

The 3D reconstructions of biocytin-stained cell morphologies were obtained from whole-cell patch-clamp experiments on 300 μm thick brain slices from juvenile mice hippocampus CA1, following experimental and post-processing procedures as previously described (Markram et al., 1997). The neurons that were chosen for 3D reconstruction were high contrast, completely stained, and had few cut arbors. Reconstruction used the Neurolucida system (MicroBrightField Inc., USA) and a bright-field light microscope (DM-6B, Olympus, Germany) at a magnification of 100× (oil immersion objective, 1.4–0.7 NA). The finest line traced at the 100× magnification with the Neurolucida program was 0.07 μm. The slice shrinkage due to staining procedure was approximately 25% in thickness (*Z*-axis). Only the shrinkage of thickness was corrected at the time of reconstruction.

### 2.3 Whole-cell patch-clamp in current clamp mode recording

The slices were placed on a recording chamber at 33°C (Temperature controller L&N) that was constantly perfused (peristaltic pump P-1, Amersham Biosciences) with oxygenated (O_2_ 95%; CO_2_ 5%) normal extracellular solution (in mM: 125.0 NaCl, 2.5 KCl, 1.0 MgCl_2_, 1.25 NaH_2_PO_4_, 2.0 CaCl_2_, 25.0 glucose, 25.0 NaHCO_3_). Neurons were located visually using an upright microscope (Olympus BX51WI) with IR-DIC optics and a digital camera (Prime 95B Teledyne photometrics). Borosilicate capillaries with filament (Hilgenberg; 1403513) were pulled with a pipette puller (p-97, Sutter Instruments) into pipettes that were filled with intracellular solution (in nM: 110.0 K-Gluconate, 10.0 KCl, 4.0 Mg-ATP, 10.0 Na-Phosphocreatine, 0.3 Na-GTP, 10.0 HEPES, 8.0 Biocytin; pH 7.3, 295 mOsm). The intracellular electrode (Ag/AgCl) presents a resistance between 5 and 10 MΩ in the bath. Somatic, current clamp, whole cell recordings were performed using Axopatch 200B amplifiers (Axon Instrument). Data acquisition was performed via ITC-18, connected to a Windows based PC (Dell), running a custom-made routine in Igor Pro (V 7.0, Wavemetrics). The voltage signal was sampled at rates between 5 and 20 kHz, and filtered with a 2 kHz Bessel filter. All chemicals were obtained from Sigma-Aldrich.

### 2.4 Statistical analysis

Results in figures are presented as box plots containing median, quartiles, minimum/maximum values and outliers, if present, or as trend of mean values ± std. error. Statistical tests were performed using built-in functions of Sigmaplot v13 (Systat Software Inc.). Datasets for mice and rats following a normal distribution were compared using a student *t*-test; Mann–Whitney Rank-sum test was used in all other cases. Multiple pairwise comparisons were not considered.

### 2.5 Feature extraction

Electrophysiological features were extracted from individual experimental traces using the feature extraction tool of the EBRAINS Cellular level modeling workflows,^1^ which uses the open-source Electrophysiological Feature Extraction Library (eFEL). For the mouse, a total of 200 traces were analyzed, using 10 different current injections. In particular, we used 6 positive injections, belonging to the protocol APwaveform, and 4 negative injections from the protocol HyperDePol. Together with the recordings previously obtained and analyzed from rats (Migliore et al., 2018), they were organized in 64 sets of recordings, with 20 neurons from mice (10 of which used in the modeling), 53 neurons from rats. For those features requiring an Action Potential threshold, a value of –20 mV was used. It should be pointed out that this threshold is different from the traditional action potential (AP) threshold calculated at the beginning of an AP. Several features require its definition, such as the action potential width (calculated at the threshold) or the spike time (calculated as the time at which there is a voltage peak within the time window defined by the threshold voltage).

### 2.6 Computational modeling

The simulations were carried out using the NEURON simulation environment [v7.7.2, (Hines and Carnevale, 1997)]. Model optimizations were performed using HPC systems, CINECA (Bologna, Italy), JSC (Juelich, Germany) or CSCS, using the open-source Blue Brain Python Optimization Library (BluePyOpt), integrated on the EBRAINS Cellular level modeling workflows (see text footnote 1) in many online use cases.

Both mouse simulations and optimizations, were implemented using a three-dimensional morphological reconstruction of the same neurons from which the corresponding traces were recorded.

Before running the cell model optimizations, *I*_*h*_ channel kinetic from Magee (1998), was fitted using the standard *Run Fitter* tool available in NEURON. The optimization was carried out simultaneously on a typical set of experimental traces and converged into a good solution, with an error lower than 0.3 mV.

All experimental and model files are publicly available on EBRAINS platform (Appukuttan et al., 2023). Complete model and simulation files will also be available on the ModelDB section of the Senselab suite.^2^

### 2.7 Model configuration

Except for the non-specific *I*_*h*_ current, separately optimized in preliminary optimization runs, channel kinetics were based on those used in many previously published papers on hippocampal neurons (Magee, 1998; Bianchi et al., 2012; Migliore et al., 2018), and validated against a number of experimental findings on CA1 pyramidal neurons. The complete set of active membrane properties included a sodium current (*Nax* or *Na3*, for axon or other compartments, respectively), four types of potassium (*K*_*DR*_, *K*_*A*_-proximal or distal for soma and axon or dendrites respectively-, *K*_*M*_, and *K*_*D*_). On soma and dendrites also three types of Calcium (*CaN*, *CaL*, *CaT*), the non-specific *I*_*h*_ current, and two types of *Ca*-dependent *K*^+^ currents (*K*_*Ca*_ and *Cagk*) were inserted. A simple Calcium extrusion mechanism, with a single exponential decay of 100 ms, was also included in all compartments containing Calcium channels. In general, channels were uniformly distributed in all dendritic compartments, except *K*_*Ap*_, *K*_*D*_ and *I*_*h*_ channels, that were arranged following an appropriate distribution as a function of distance from the soma (Migliore et al., 2018). The values for the peak conductance of each channel were independently optimized in each type of compartment (soma, axon, basal and apical dendrites). The parameters’ range was defined with preliminary simulations, and it covered a range of at least one order of magnitude.

All simulations were performed by activating excitatory synapses targeting the oblique dendrites, representing the excitatory Schaffer Collateral afferent pathway from CA3 to CA1 pyramidal neuron dendrites in the Stratum Radiatum. Following experimental findings (Megías et al., 2001; Bezaire and Soltesz, 2013) and considering the morphological features of rat and mouse, we randomly distributed a set of CA3-CA1 AMPA synapses in the thinner apical dendrites (smaller then 1.2 for rat and 1 μm for mouse) within 360 (rat) and 330 μm (mouse) from the soma. All synapses were modeled using the NEURON class Exp2Syn(). Peak conductances, consistent with published excitatory postsynaptic current (EPSCs) amplitudes (Magee and Cook, 2000; Andrásfalvy and Mody, 2006), were initially set at 0.25 nS (the average of the values for proximal, middle and distal regions for both mouse and rat calculated from the amplitude of evoked EPSCs using the Ohm law). Unless otherwise noted, 80 synapses (mimicking the population of synapses that could be activated by any given physiological stimulus) were used for both rat and mouse, with the synaptic activation patterns that will be discussed later (see section “3.4 Response to synaptic inputs”).

## 3 Results

**FIGURE 1:**
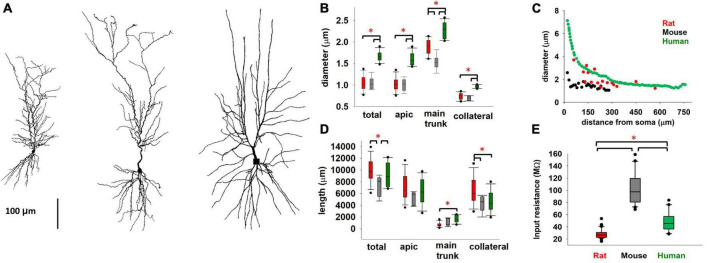
**(A)** Comparison between typical mouse (left), rat (middle), and human (right, from Benavides-Piccione et al., 2020) CA1 pyramidal morphologies. **(B)** Diameter of different type of dendritic processes **(C)** Apical trunk diameter, as a function of the distance from soma, from a typical mouse, rat, and human neuron. **(D)** Total length of different type of dendritic processes. **(E)** Input resistance of rat, mouse, and human CA1 pyramidal neurons. Rat data are taken from Migliore et al. (2018); human data taken and redrawn from Benavides-Piccione et al. (2020), and Deitcher et al. (2017), for morphological properties and R_in_ respectively. Dataset used in panels **(B,D)** contains 6, 12, and 11 cells for mice, rats, and humans respectively. Dataset used in panel **(E)** contains 20, 57, and 24 recordings for mice, rats, and humans respectively. Bars and * represent values with statistically significant differences (*p* ≤ 0.05).

We begin by comparing diameter and length of the different dendritic processes of mouse, rat, and human (Benavides-Piccione et al., 2020) CA1 pyramidal neurons. Typical morphologies are plotted in Figure 1A. From a general point of view, neuron size and branching patterns can change with age and across cortical areas (Zhang, 2004; Elston and Fujita, 2014; Deitcher et al., 2017; Gilman et al., 2017). Little differences have been found, in previous literature reports, comparing adult C57BL/6 mice and Sprague–Dawley rats (Routh et al., 2009, 5–7-week-old animals). Here, we found (Figure 1B) that human neurons, from men 20 to 57 years old, have a systematically larger dendritic diameter, with respect to both mouse and rat. Interestingly, while thinner dendritic processes (such as apical and basal dendrites) exhibit the same overall diameter in mice and rats, their main apical trunk is statistically different, with the rats having larger trunks, and thus closer to those measured in human neurons. For young rats (P14-23), as in our case (Figure 1B), we found that the average diameter is larger (and more similar to human), with respect to older mice reported by Routh et al. (2009). This is interesting because the main apical trunk diameter is an important determinant of signal propagation over the entire neuron. This trend, i.e., a diameter increase of the main apical trunk from mouse to rat and human, was consistently true also with respect to diameter tapering as a function of distance from the soma, as shown in Figure 1C. Another important determinant of a neuron excitability profile is the total dendritic length (correlated to the total neuron capacitance). In Figure 1D we report the total and differential dendritic length for the three species.

The overall result for the total dendritic length suggested that there are not statistically significant differences between rat and human neurons, whereas they are both significantly different from mouse. One of the consequences of these differences is reflected in the cell’s input resistance, R_*in*_, reported in Figure 1E. For rats and mice, we calculated R_*in*_ based on Ohm’s law using the steady-state voltage achieved during the smallest negative somatic current injection that was used in the experimental protocol, whereas for humans it was extracted from the data reported by Deitcher et al. (2017). These results suggest a similarity between rat and human neurons, with respect to mouse. We are aware that a significant contribution to the difference between mouse and rat could derive from the different protocol used for the recordings (patch-clamp or sharp microelectrode). However, we think the pronounced 3-fold difference in input resistance suggests that there may be additional contributing factors, both dependent on the morphological differences discussed above and intrinsic active and passive membrane properties. This is also consistent with experimental findings suggesting that the distribution of somatodendritic ion channels may lead to differences between species (Kalmbach et al., 2018). For this reason, we performed a more detailed comparison of the electrophysiological properties.

### 3.1 Comparison of electrophysiological features from rat and mouse recordings

**FIGURE 2:**
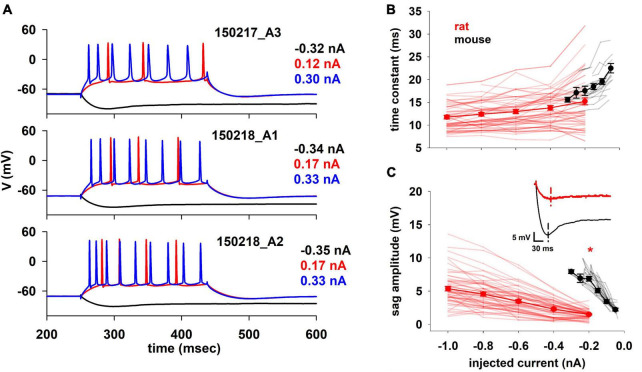
**(A)** Typical experimental voltage traces for mouse CA1 pyramidal cells during stimulation protocol at selected input current values. **(B)** Membrane time constant and **(C)** Sag amplitude trends of rat and mouse as function of injected current. In the inset typical recordings for a –0.2 nA current injection. Dotted lines indicate the timing when the minimum voltage was reached. Results for individual neurons are plotted with thin lines, mean values (±sem) are plotted as closed circles and thick lines. The red marker indicates currents with statistically significantly different results (*p* ≤ 0.05). Dataset used contains 20 and 57 recordings for mice and rats respectively.

For mouse analysis and optimization we used new, previously unpublished, datasets whereas for rats we used the same traces as in Migliore et al. (2018). Differently from morphology, for which both sets of experiments were conducted simultaneously in the same lab using identical protocols, solutions, and histological cuts, the electrophysiological recordings are from different laboratories and were recorded using different protocols. Despite this, supported by literature reports demonstrating that in frog tadpoles the major electrophysiological behavior is not significantly affected by the acquisition technique (patch clamp or sharp microelectrode) (Li et al., 2004), we decided to proceed with the analysis. A few representative traces from mice are shown in Figure 2A. Under a constant somatic current injection, they exhibited a regular firing pattern, which in most cases was weakly adapting. For hyperpolarizing currents (black traces in Figure 2A), a prominent sag was present in all neurons, a clear indication that in mice hippocampal CA1 pyramidal neurons an *I*_*h*_ current is systematically expressed in the membrane. Unless otherwise noted, all the electrophysiological features were extracted using the NeuroFeatureExtractor, a free open source tool available on the EBRAINS Infrastructure (see text footnote 1), (Bologna et al., 2021). The full list of features is reported in Supplementary Tables 2–4. In the following paragraphs, we discuss all features related to the passive properties, firing frequency, and action potential (AP) shape that appeared to better characterize the differences. In general, the rationale for restricting the features to analyze was to exert extra caution, and avoid specific speculations, in comparing features that are strongly dependent on ionic channel kinetics, for which we don’t have sufficient experimental information on the possible interspecies differences.

We first examined two basic electrophysiological properties, the membrane time constant (Figure 2B) and the sag amplitude (Figure 2C), as a function of injected current. The membrane time constant plays an important role in signal propagation and integration (Rall, 1977), which can also be strongly modulated by *I*_*h*_ (Magee, 1999). In our case, the time constant was calculated by fitting a single exponential curve to the initial profile of the membrane potential during a hyperpolarizing current. In principle, the intrinsic time constant of the membrane should be independent of the injected current. However, the presence of the *I*_*h*_, which is already active at rest and is increasingly activated by hyperpolarization, causes the measured time constant to decrease with hyperpolarization. This effect is much smaller in rats than in mice, where the time constant decreases more rapidly with the injected current (22.74 and 15.72 ms/nA, for mouse and rat respectively).

The sag amplitude (Figure 2C) is a direct consequence of the *I*_*h*_ (e.g., Magee, 1999). We found a significant difference in its amplitude between rats and mice. Although the different experimental range and protocol used to record from mice and rats allowed a direct statistical comparison only at −0.2 nA (*p* < 0.001), the results are clear for the entire current range. A linear fit of the mean values indicated that the two species respond differently to an input (1.33 and 0.43 mV/nA, for mouse and rat respectively). This current has a prominent role in synaptic integration, especially in rat hippocampal CA1 pyramidal neurons, where its dendritic expression increases with distance from the soma (Magee, 2001). To the best of our knowledge, it is not experimentally known if this peculiar distribution is valid also for mice, but these results suggest a similar trend.

**FIGURE 3:**
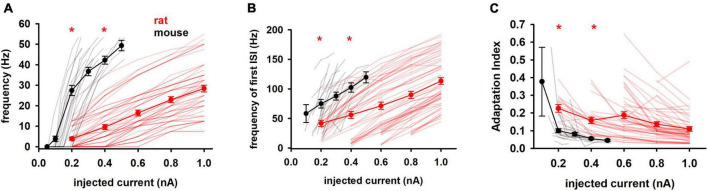
Firing features **(A)** Mean frequency, **(B)** frequency of first ISI and **(C)** adaptation index of rat and mouse as function of injected current. Results for individual neurons are plotted with thin lines, mean values (±sem) are plotted as closed circles and thick lines. Red markers indicate currents with statistically significantly different results (*p* ≤ 0.05). Dataset used contains 20 and 57 recordings for mice and rats respectively.

We then considered features related to the firing profile of the cells, which is related to the way in which information is processed in the brain. Since it has been reported that in the rat hippocampus neurons characterized by different firing patterns have different physiological and morphological identities (Graves et al., 2012), the analysis of these parameters could provide important information about the differences between the two species. In particular, the number of APs and their distribution over the stimulation period are very important for signal transmission in the network. As expected from the large difference in the input resistance, we found that electrophysiological properties based on firing behavior showed significant statistical differences between mouse and rat cells, as a function of the input current, as shown in Figure 3. Mouse cells fired at a much higher frequency than rat cells (Figure 3A) and, on average, they begin to show a response saturation at relatively low currents, whereas the average rat response curve shows no signs of saturation up to 1 nA. Also striking in both species are the wide range of currents to generate at least one AP (rheobase), which can be appreciated in Figure 3A as the first current for which the frequency is >0.

As shown in Figure 3B, mouse neurons also fired at an instantaneous frequency higher than rat neurons and increasing more rapidly with input current (149.7 Hz/nA vs 88.61 Hz/nA in mice and rats, respectively). Finally, the adaptation index (Figure 3C) was much lower in mice, although its slope was quite similar in both species (−0.19 and −0.13 in mice and rats, respectively). This suggests that neurons from mice and rats respond similarly to changes in input current, although adaptation differs during a train.

Next, we considered the shape-related features of APs. It is known that the kinetics of an action potential are modulated by sodium and fast-gating potassium currents, with calcium and calcium-dependent potassium currents also contributing. Changes in AP-related features may therefore provide information on whether there are significant changes in the expression of these channels in the two species.

**FIGURE 4:**
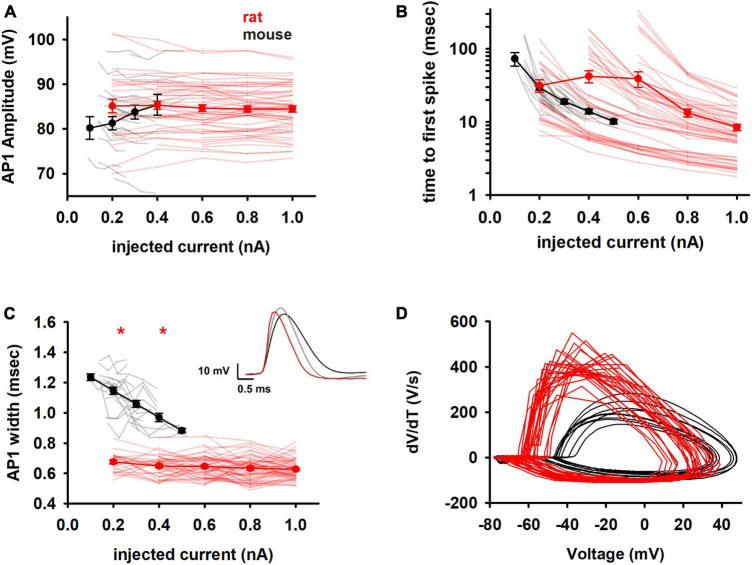
Spike shape features **(A)** amplitude and **(B)** time of the first action potential in the train of rat and mouse as function of injected current. Y axis is reported as log scale. **(C)** Width of the first spike. In the inset comparison of typical first APs of mouse at 0.2 nA (black) and 0.4 nA (gray), and rat at 0.4 nA (red). **(D)** Phase plots for the first AP for a 0.2 nA current injection. Results for individual neurons are plotted with thin lines, mean values (±sem) are plotted as closed circles and thick lines. Red markers indicate currents with statistically significantly different results (*p* ≤ 0.05). Dataset used contains 20 and 57 recordings for mice and rats respectively.

The amplitude of the first AP was highly variable and similar in both species (Figure 4A). The time to the first spike (Figure 4B), which gives information on the type of channels open near rest, also showed a large variability for rats (over one order of magnitude at any current) and made it impossible to distinguish between the two species. In contrast, the width of the first AP (Figure 4C) showed significant differences in its absolute value and trend with increasing current injection. Notably, mouse neurons were characterized by a much broader AP width that decreased sharply with increasing depolarizing currents. In rats, the first AP was shorter and essentially maintained its value over the entire range of currents used (compare black and red lines in Figure 4C). Since the experiments were performed at the same temperature, the shorter AP in the rat cannot be caused by a temperature-dependent reduction of the time constant for activation/inactivation kinetics; something else, such as a different interplay between Na^+^ and K^+^ channels can be involved. The qualitative difference in the AP shape in the two species is evident by the phase plots shown in Figure 4D.

### 3.2 Computational model of individual cells

We used the results discussed above to carry out a data-driven implementation of mice CA1 pyramidal neurons. As for the rat (Migliore et al., 2018), except for the *I*_*h*_ kinetics, we limited the features used to optimize the models to those directly related to the spike times. The rationale for this choice was that, to the best of our knowledge, there is not enough experimental information on the differences between ion channel kinetics in mice and rat hippocampal neurons, that would help in implementing more species-specific computational models. However, given that the spike times indirectly reflect these differences, we considered this a plausible effective choice.

**FIGURE 5:**
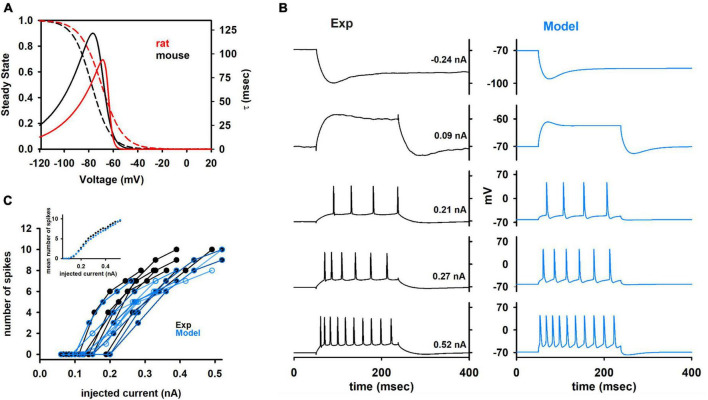
**(A)** Comparison between the I_h_ kinetic for rat and mouse. Dotted lines represent steady state and solid lines represent the time constant of activation. List of fitting Parameters (values are indicated in black for mouse and in red for rat): Vl_1/2_ = –77.46 and –69.5 mV; Vt_1/2_ = –70.24 and –64.1 mV; a0t = 4.7 × 10^–3^ and 7.2 × 10^–3^ ms^–1^; zetal = 3.5 and 5.2; zetat = 7.3 and 15.8; gmt = 0.145 and 0.067; clk = 0.24 and 1. **(B)** Comparison between typical experimental and model traces at different somatic current injection values. **(C)** Number of spikes as a function of the input current from experiments (black traces) and models (blue traces). Different cells have different set of values for input current. Therefore, averaging the number of spikes for all cells at different values of input current is not straightforward. The inset shows the average results for experiments and model.

We started from the results obtained from preliminary simulations, suggesting that the kinetics of the *I*_*h*_ current, as reported in the literature and optimized for rats (Migliore and Migliore, 2012) was not able to fit both the sag and the membrane time constant seen in the experiments (data not shown). For this reason, following the same fitting procedure described in Vitale et al. (2021), we implemented a new *I*_*h*_ current model specifically based on the data set used in this work, also including passive membrane properties as fitting parameters. The optimized kinetic is compared in Figure 5A with that used for rat neurons, and it suggests that the *I*_*h*_ in mouse CA1 hippocampal neurons can be up to 4 times slower than in rat neurons, with negligible changes in the steady-state activation curve.

For the full model optimization, we used the BluePyOpt tool (Van Geit et al., 2016) embedded into a workflow available on the EBRAINS infrastructure (Bologna et al., 2022), and in interactive use cases (see text footnote 1). BluePyOpt, is a multi-objective genetic algorithm that returns several suitable ensembles model parameters, in our case the peak ion channel conductances and passive properties, which best fit the experimental features. The main criterion used to accept an ensemble of parameters to model a given cell (called “an individual”) was based on the standard deviation (sd) of the fit obtained for each considered feature. An individual with a sd < 3 for all features was considered acceptable (a sd = 0 corresponds to a perfect match between model and experiment). The procedure generated a population of individuals mimicking the intrinsic physiological variability of the neurons considered in this paper.

It should be noted that, in contrast to our previous work with rat traces (Migliore et al., 2018), where we have implemented models based on average features, calculated as a function of the input current over the whole dataset, here the feature extraction and optimization was performed for each set of experimental traces from any given cell. It is important to note that the variability obtained in the rat’s optimizations covers the entire observed experimental range of excitability properties, and that the rat dataset is characterized by less variability in single-cell behavior with respect to mice. For this reason, we are confident that the models obtained from the two approaches can be suitably compared. The model for each mouse neuron was optimized using also the morphology reconstructed after the recordings. The use of traces and morphology from the same neuron allows to better highlight the intrinsic properties of different neurons. The set of features that were considered for the optimization procedure are shown in Table 1.

**TABLE 1:** Optimized features.

Voltage features	Spike event features
Voltage base steady_state_voltage voltage_deflection sag_amplitude	Spikecount_stimint time_to_first_spike time_to_last_spike inv_first_ISI inv_second_ISI inv_third_ISI inv_fourth_ISI inv_fifth_ISI inv_last_ISI

List of features used to optimize mouse models. Please refer to the EFEL documentation for name and explanation of each feature (https://pypi.org/project/efel/).

In Figure 5B we compare a typical optimization result with the experimental recordings from cell 150217_A3. In Figure 5C the results in terms of the number of spikes as a function of the input current are compared with experimental findings from all cells. As can be seen, the models were in good agreement with experiments, and reproduced the large variability observed experimentally. The average number of spikes elicited as a function of the input current (Figure 5C, inset) was statistically indistinguishable from those obtained from experiments (*p* = 0.847). A good representation of the spike times as a function of the input current is a particularly important result, since it is one of the most important features to consider for large, full-scale, hippocampal network models.

### 3.3 Channel distribution and degeneracy. An analysis across species

As mentioned in the previous section, the experimental variability observed among individual cells belonging to a given homogeneous cells population can be related to the different expression of membrane channels. It is the basis for neuronal degeneracy, which is the capability to adapt and adjust input/output properties using structurally different elements. This allows individual neurons to maintain the appropriate and robust functionality of a brain network, even in the presence of protein turn-over, small injuries, or weak pathological conditions (Edelman and Gally, 2001; Migliore and Shepherd, 2002; Drion et al., 2015).

**FIGURE 6:**
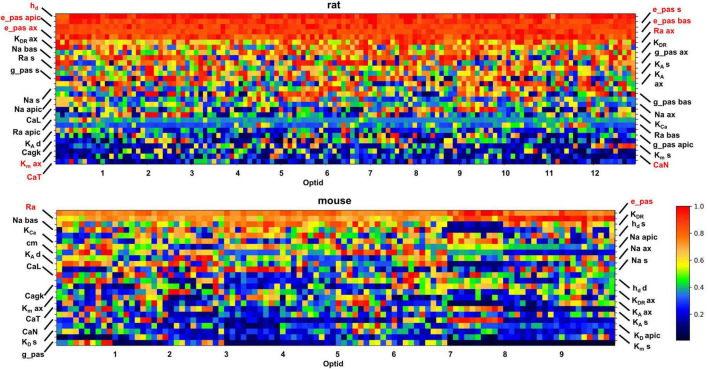
Set of parameters describing the modeled cells, normalized to the maximum value, obtained from the 10 best individuals from each optimization (10 for mice and 13 for rats). The *X*-axis reports the ID of each cell, followed by its 10 best individuals. On the *Y*-axis the parameters are reported. The pixel color is related to the value of a given parameter, based on the color scale on the right.

The optimization process generated for each target set of experimental traces not only the best individual (i.e., the set of parameters that best reproduces the experimental traces) but also many additional individuals that are essentially equally good (i.e., with an overall error slightly higher than the best one). This gives the possibility to better understand the differences between rat and mouse neurons at the membrane properties level. For this purpose, we compared the optimized models in trying to identify those parameters which contribute to the firing properties of the studied cells. In Figure 6 we report normalized values of the optimized parameters (*Y*-axis), for the 10 best individuals of each optimized cell (in the *X*-axis, 13 for rats and 10 for mice). Parameter values are normalized to their maximum and sorted on the *Y* axis according to their average value. In this way, the upper rows correspond to parameters with higher average values whereas the lower ones correspond to parameters with lower average values.

Inspection of Figure 6 indicates that most of the parameters can assume a wide range of values, as shown by the different colors in most rows in the graphs for the two species. In other words, it means that the physiological behavior of the CA1 pyramidal neurons can be equally well reproduced by combining the set of channels’ peak conductance in a relatively large number of ways. This phenomenon is related to the ability of neurons to easily tune and adapt their response during their lifespan. The models appear to capture it well, with many optimizations (individuals) fitting equally well the set of reference traces with a rather different combination of channel conductances. It should be noted that the parameters that are relatively stable over the different optimizations are related to electrophysiological properties that must be expressed at a constant level throughout the neuronal population, to avoid significant changes in the firing properties. For rats, as discussed in Migliore et al. (2018), the passive properties, the *I*_*h*_, *K*_*M*_, and some type of Calcium channels appears to be preferentially expressed at a relatively high or low density in all neurons (see top panel in Figure 6). In mouse this effect is much less evident and restricted to only a few passive properties (shown in red in the bottom panel of Figure 6). Interestingly, studies of temporal lobe epilepsy have shown that changes in *I*_*h*_ and *K*_*M*_ correlates with altered excitability (Poolos et al., 2002; Miceli et al., 2013). For this reason, the model suggests that rats may be the species of choice when studying this brain disease.

**FIGURE 7:**
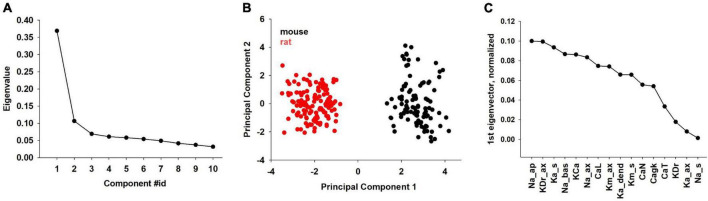
Principal component analysis performed on the subset of common mouse and rat parameters. **(A)** Contributions to the overall variance in parameter space (eigenvalues) of the first ten PCA components. **(B)** Projection of all mouse (black dots) and rat (red dots) neurons onto the first two PCA components. **(C)** Individual contribution to the first PCA component of each parameter.

Further insight on species-specific characteristics should result from a direct comparison of the combination of individual ion channels in rats and mice. To this aim, we performed principal component analysis (PCA) on the subset of parameters common to mice and rats (Figure 7). The obtained principal components are collective variables (linear independent combinations of the parameters) and are ordered by decreasing variance. The first ten PCA components account for almost 90% of the variance of the parameters (Figure 7A). Surprisingly, the data projection on the first PCA component clearly separates the two species into two distinct clusters (Figure 7B), while all other projections do not. The individual contribution of each parameter to the first PCA component is shown in Figure 7C.

Among the parameters whose effect is largest on the first PCA component (i.e., more strongly contributing to the differences between rat and mouse models) we found the dendritic Na, axonal *K*_*Dr*_, somatic *K*_*A*_ and the Calcium dependent K-current. Interestingly, these parameters are directly related to neuron excitability, adaptation, and AP duration. These are also the features that showed statistical differences in the analysis of the experimental recordings (see Figures 3, 4), and the model thus makes the experimentally testable prediction that these channels would be expressed at a significantly different density in mouse and rat CA1 pyramidal neurons.

### 3.4 Response to synaptic inputs

In the previous sections we analyzed the inter-species differences in terms of electrophysiological features and firing patterns in response to somatic current injections.

**FIGURE 8:**
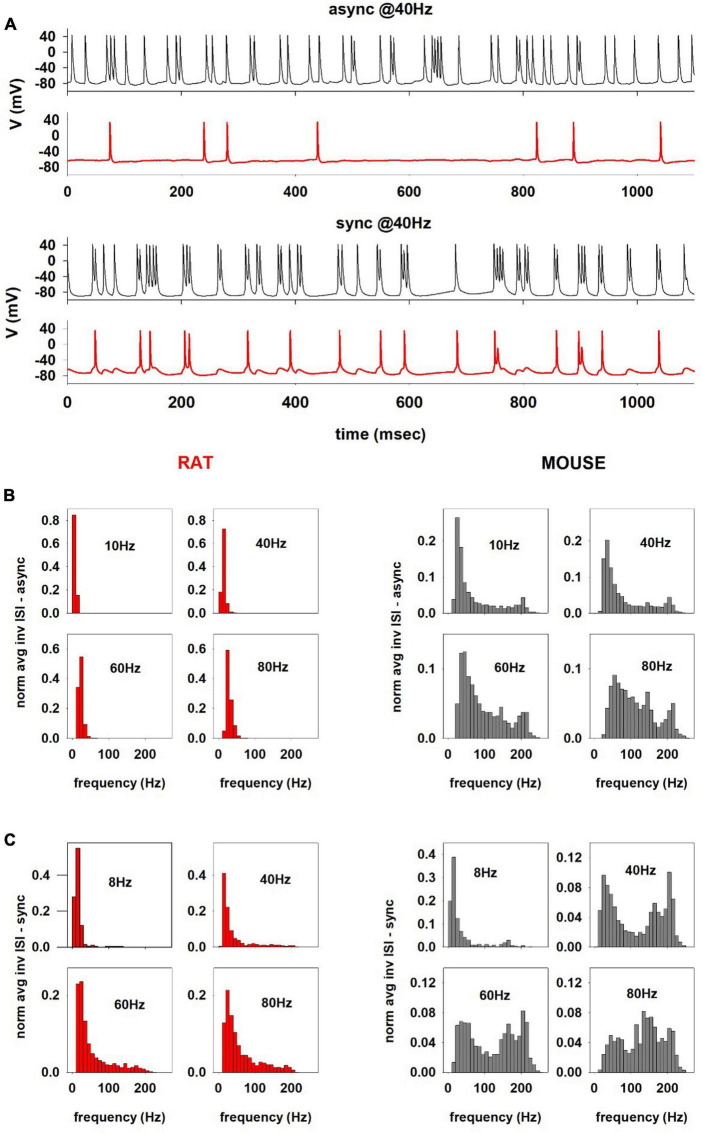
**(A)** Comparison between the firing activity charactering the hippocampal CA1 computational models of mouse (cell 150217_A3_idB, black traces) and rat (cell oh140807_A0_idG, red traces) for 40 Hz asynchronous (top) and synchronous (bottom) inputs. Distributions of the instantaneous AP firing frequency (calculated as the reciprocal of the Inter-Spike Intervals, ISI^−1^) for different average activation frequency for asynchronous **(B)** and synchronous synaptic inputs for the rat and mouse **(C)**.

To gain further insight into the behavior of these neurons under more *in vivo* conditions, we next compared a mouse and rat model response to synaptic activations. For this purpose, a set of simulations (*n* = 17) was carried out for a mouse and rat cell (using the optimized models for 150217_A3_idB and oh140807_A0_idG, respectively) using 80 excitatory synaptic inputs activated at an average (Poisson) frequency in the *theta* (8 or 10 Hz) and *gamma* range (40, 60, and 80 Hz), activated synchronously or asynchronously, in such a way to consider the widely different activity that can be expected from cognitive processes in the oblique apical dendrites. Synapses where randomly distributed in the oblique dendrites, where most of the Schaeffer Collaterals from CA3 make their synaptic contacts (Megías et al., 2001). Typical simulated traces for 40 Hz inputs are shown in Figure 8A; the difference between mouse (Figure 8A, black traces) and rat (Figure 8A, red traces) are evident, with mouse recordings exhibiting a much stronger and heterogeneous (bursting) activity, with respect to what observed in rat, where activity was mainly formed by single spikes with occasional doublets.

To better characterize the difference, also as a function of the input, in Figure 8B we plot the distributions of the instantaneous AP firing frequency (calculated as the reciprocal of the Inter-Spike Intervals, ISI^−1^) as a function of the average activation frequency of asynchronous synaptic inputs. For the rat neuron (Figure 8B, red plots), at all frequencies the ISI distributions were rather narrow and with little or no components higher than the stimulation frequency. In contrast, in the mouse neuron (Figure 8B, gray plots) the distributions were wider and included instantaneous frequencies up to approximately 250 Hz.

For synchronous inputs (Figure 8C), the difference between mouse and rat neurons was even more striking, especially over the entire *gamma* range (Figure 8C, 40, 60, and 80 Hz); the mouse neuron exhibited a bimodal distribution with a significant component at 150–250 Hz, i.e., in the sharp wave-ripple (SWR) range, which was essentially independent from the stimulation frequency. The distribution obtained from the rat’s neuron did not show the same behavior at any frequency (Figure 8B, lower plots), although the distributions were wider than those for the asynchronous input. Interestingly, experimental findings suggested that SWR activity is associated with synchronous hippocampal cell firing mediating memory retrieval processes (Joo and Frank, 2018). However, since SWRs are also observed in rats (Aleman-Zapata et al., 2022), we investigated the reason why our model did not reproduce them. We noticed that high-frequency ISIs in the mouse correspond to a high occurrence of short AP bursts during the stimulation, which were rarely observed in the rat neuron. A plausible explanation for the presence of SWR frequencies in the mouse could thus be the high input resistance of the (thin) apical dendrites, which can result in a strong dendritic depolarizing envelope (more easily than in the relatively thicker rat’s dendrites) in response to overlapping synaptic inputs; this effect would more easily produce a burst of somatic APs. To test this hypothesis, we ran a rat simulation using a 3-fold increase of the synaptic weight synchronously activated at 80 Hz. The rationale for this test was that a higher synaptic conductance would generate the type of stronger local depolarization that is required to elicit bursts of APs. The results, show in Supplementary Figure 1, confirmed the appearance of a high-frequency component in the ISIs. The model points out that the same synaptic input can trigger this firing modality in mice (but not rat) hippocampal neurons, in response to synchronous CA3 synaptic inputs in the *gamma* range. We also carried out an additional set of simulations with a more realistic representation of synaptic activity, in which bursts of synaptic inputs in the Gamma rhythm range were activated at an average frequency in the Theta range, to mimic the burst of activity typically observed *in vivo* from CA1 pyramidal neurons during cognitive processes. The results (Supplementary Figure 2) confirmed those obtained with the stationary Poisson process.

Taken together these results point out to a significant difference, between mouse and rat hippocampal pyramidal neurons, in the response to synaptic inputs at behaviorally relevant frequencies.

## 4 Discussion

By analyzing experimental data and simulation findings from young mice and rats CA1 pyramidal neurons, we found that there are significant differences in the way in which they respond to artificial or behaviorally relevant stimuli. Overall, the results suggest that in the studied population, rat neurons are more similar to human neurons, with respect to mice neurons. This aspect is usually not considered in extrapolating experimental findings to humans.

Analysis of morphological and electrophysiological properties of same age mice and rats, suggested that the total, but also the local, input resistance appears to be a major determinant of the differences in the electrophysiological behaviors under both *in vitro* (i.e., constant current injection) and *in vivo* (i.e., synaptic inputs) conditions. The same somatic constant current injection generates in the mouse a much higher mean and instantaneous frequency, and a different adaptation index profile as a function of the injected current, with respect to rat (Figure 3). Several other differences in the electrophysiological features can be explained by different ion channel properties and/or distribution. Although it has been suggested that the kinetic properties of ion channel in the neuronal membrane of mammals are conserved across mouse, rat, and human [at least for Kv channels, (Ranjan et al., 2019)], their actual density and kinetic properties can be continuously modulated by a number of activity-dependent biochemical pathways (e.g., Johnston et al., 2000) and by degeneracy (Migliore et al., 2018). From this point of view, we found that the AP shape (which can be modulated by the sodium and potassium channel kinetics and interplay) was characterized in mice neurons by a broader AP that decreased sharply with increasing depolarizing currents, in contrast with the rather constant value over the entire range of the injected currents in rats (Figure 4C). Furthermore, mice also exhibited a significantly slower *I*_*h*_, with respect to rats, implying different synaptic integration properties (Bittner et al., 2012).

Computational models for individual (*n* = 10) mouse neurons, able to reproduce the I/O profile each cell (Figure 5C) and their large experimental variability (Figure 5B), allowed us to obtain a quantitative estimation of the expected range of ionic channel conductance and dendritic distribution parameters, and to perform a systematic comparison between the two species under *in vivo*-like conditions. For both mice and rats, the electrophysiological activity of a given neuron can be reproduced with very different ensemble of values for the peak channels’ conductance. It is interesting to note that the more sensitive parameters (that are presumably related to pathologies) are different for mice and rats. We have previously found (Migliore et al., 2018) that in rats the *I*_*h*_, *K*_*M*_, and calcium *CaN* and *CaT* channels need to be constrained within a limited range of values to reproduce physiological traces. In contrast, only the membrane passive properties need to be constrained in mice. Such a finding suggests that whereas mice would be much more resistant to mutations affecting their (hippocampal) ionic channels, rat offers many advantages over the mouse as a model of human diseases such as epilepsy, which it has been strongly correlated to mutations in the *I*_*h*_ (Poolos et al., 2002) and *K*_*M*_ (Miceli et al., 2013) currents. A principal component analysis on mouse and rat model parameters (Figure 7), revealed that the two species can be grouped into two well distinguishable clusters, with the dendritic *Na*^+^, the axonal *K*_*Dr*_ the somatic *K*_*A*_ and the *K*_*Ca*_ as major determinants of their differences.

Intriguingly, the model results pointed out differences also under *in vivo*-like conditions, in response to synchronous and asynchronous synaptic activations in the *theta* and *gamma* range (Figure 8). These conditions cannot be easily studied experimentally because it is essentially impossible to adequately control synaptic inputs *in vivo*. Assuming, as suggested experimentally (Magee and Cook, 2000; Andrásfalvy and Mody, 2006), that individual synaptic properties and number are similar for the two species, we found that a mouse would exhibit an activity in the range of hippocampal Sharp-Wave Ripples (SWRs), through a much stronger high-frequency bursting activity with respect to the rat. Since SWRs have been shown to be involved in memory consolidation processes (Buzsáki, 2015), this result suggest a physiological plausible reason for the observed behavioral differences between mice and rats (Frick et al., 2000). Interestingly, to obtain in a rat results comparable with those obtained in a mouse, the model predicts that the synaptic input should be at least 3 times stronger. This implies more (synchronous) synapses, and thus a higher connectivity, or a higher individual conductance. Given that experimental findings indicated that the peak conductance of individual synapses is in the same range for rat and mouse hippocampus (Magee and Cook, 2000, for rats, Andrásfalvy and Mody, 2006, for mice), and that SWR are observed in the rat too (Joo and Frank, 2018), these results suggest that a rat CA1 pyramidal neuron should receive approximately 3 times more CA3 inputs with respect to mouse.

In conclusion, our findings: (1) support the notion that “*Mice are not little rats*” (Frick et al., 2000), (2) suggest an electrophysiological basis for their differences, (3) point out the reasons why rats should be preferred over mice for studying the human brain at different scales of integration and complexity, at least referring to the hippocampal CA1 principal neurons, and (4) suggest that the choice of a specific rodent model should also depend on the specific property or behavior to be investigated.

## Data availability statement

The datasets presented in this study can be found in online repositories. The names of the repository/repositories and accession number(s) can be found below: Data and models will be available at the live paper section of ebrains https://ebrains.eu/services/data-and-knowledge. Model will be also available on Model DB https://modeldb.science/2014816.

## Ethics statement

The animal study was approved by the Swiss Cantonal Veterinary Office Committee for Animal Experimentation. The study was conducted in accordance with the local legislation and institutional requirements.

## Author contributions

PV: Conceptualization, Formal analysis, Investigation, Writing – original draft. FL: Formal analysis, Investigation, Writing – review and editing. AV: Formal analysis, Investigation, Writing – review and editing. EC: Investigation, Writing – review and editing. MP: Data curation, Investigation, Writing – review and editing. YS: Data curation, Investigation, Writing – review and editing. AR: Data curation, Investigation, Writing – review and editing. MM: Conceptualization, Funding acquisition, Investigation, Project administration, Supervision, Writing – original draft. RM: Conceptualization, Formal analysis, Investigation, Writing – original draft.

## Footnotes

1
https://ebrains.eu/service/cls-interactive/

2
https://modeldb.science/2014816
