# Supplementary material for: Different responses of mice and rats hippocampus CA1 pyramidal neurons to in vitro and in vivo-like inputs
Source: Front Cell Neurosci. 2023 Dec 7;17:1281932. doi: 10.3389/fncel.2023.1281932 (PMC10733970; doi:10.3389/fncel.2023.1281932)
Supplement: Supplementary file 1 [file Data_Sheet_1.docx]

***Vitale et al. - Supplementary Materials***

Different responses of mice and rats hippocampus CA1 pyramidal neurons to *in vitro* and *in vivo*-like inputs

**Table S1: Comparison between experimental protocols**

|  |  | **Mouse** | **Rat^1^** | | **Human^2^** |
| --- | --- | --- | --- | --- | --- |
|  |  | Morph. & Ephys | Morph. | Ephys | Morph |
| **age** |  | P 13-16 | P 14-23 | Weight 90-180  (about 3.5-5.5 W) | 20-57 y |
| **strain** |  | C57BL/6J | Wistar | Sprague Dawely | - |
| **Temperature (°C)** |  | 33 | 34 ± 1 | 34-36 | 32-35 |
| **Cuts thickness (μm)** |  | 300 | 300 | 450-500 | 300-400 |
| **aCSF solution** | Na^+^  K^+^  Ca^2+^  Mg^2+^  Total negative ions (mM) | 151.25  2.5  2  1  159.75 | 149.5  4.5  2.5  1  160 | 149.5  4.5  2.5  1  160 | 153  3  2  1  161 |
| **Pipette solution** | Na^+^  K^+^  Mg^2+^  Total negative ions (mM) | 10.3  120  4  134.3 | 10.3  120  4  134.3 | - | 0.4  140  4  134.3 |
| **pH** |  | 7.3 | 7.3 | - | 7.3 |
| **mOsm** |  | 295 | 290-300 | - | 280-290 |
| **References:** ^1^ Migliore, R., Lupascu, C. A., Bologna, L. L., Romani, A., Courcol, J.-D., Antonel, S., et al. (2018). The physiological variability of channel density in hippocampal CA1 pyramidal cells and interneurons explored using a unified data-driven modeling workflow. PLOS Computational Biology 14, e1006423. doi: 10.1371/journal.pcbi.1006423.  ^2^ Deitcher, Y., Eyal, G., Kanari, L., Verhoog, M. B., Atenekeng Kahou, G. A., Mansvelder, H. D., et al. (2017). Comprehensive Morpho-Electrotonic Analysis Shows 2 Distinct Classes of L2 and L3 Pyramidal Neurons in Human Temporal Cortex. Cerebral Cortex 27, 5398–5414. doi: 10.1093/cercor/bhx226. | | | | | |

**Table S2: Passive membrane properties**

| **Features name** | **Current (nA)** | **mouse** | **Current (nA)** | **rat** | ***p*** |
| --- | --- | --- | --- | --- | --- |
| **Input resistance**  **(MΩ)** | - | **86 ± 3** | - | **28 ± 1** | **<0.001** |
| **Time constant**  **(ms)** | -0.30  -0.25  -0.20  -0.15  -0.10  -0.05 | 15.6 ± 0.5  17.1 ± 1.2  17.5 ± 1.0  18.5 ± 0.6  19.6 ± 0.6  22.5 ± 1.0 | -1.00  -0.80  -0.60  -0.40  -0.20 | 11.8 ± 0.4  12.4 ± 0.4  13.0 ± 0.5  13.8 ± 0.5  15.2 ± 0.8 | 0.214 (@ -0.2nA) |
| **Sag amplitude**  **(mV)** | -0.30  -0.25  **-0.20**  -0.15  -0.10  -0.05 | 7.9 ± 0.3  6.9 ± 0.6  **6.8 ± 0.3**  5.1 ± 0.3  3.4 ± 0.3  2.2 ± 0.2 | -1.00  -0.80  -0.60  -0.40  **-0.20** | 5.4 ± 0.3  4.6 ± 0.3  3.5 ± 0.2  2.3 ± 0.2  **1.5 ± 0.1** | **<0.001 (@ -0.2nA)** |

**Table S3: First AP features**

| **Features name** | **Current**  **(nA)** | **mouse** | **Current**  **(nA)** | **rat** | ***p*** |
| --- | --- | --- | --- | --- | --- |
| **Time to**  **first spike**  **(ms)** | 0.10  0.20  0.30  0.40  0.50 | 73.5 ± 15.6  29.3 ± 2.2  19.0 ± 1.5  14.0 ± 1.1  10.2 ± 0.8 | 0.20  0.40  0.60  0.80  1.00 | 31.6 ± 6.2  42.2 ± 7.8  39.1 ± 9.3  13.4 ± 1.7  8.5 ± 0.9 | 0.104 (@ 0.2nA) 0.572 (@ 0.4nA) |
| **frequency**  **of first ISI**  **(Hz)** | 0.10  **0.20**  0.30  **0.40**  0.50 | 58 ± 15  **75 ± 7**  88 ± 7  **102 ± 8**  120 ± 10 | **0.20**  **0.40**  0.60  0.80  1.00 | **42 ± 5**  **56 ± 6**  71 ± 6  90 ± 6  113 ± 6 | **0.002 (@ 0.2nA) <0.001 (@ 0.4nA)** |
| **First**  **AP width**  **(ms)** | 0.10  **0.20**  0.30  **0.40**  0.50 | 1.24 ± 0.02  **1.15 ± 0.02**  1.06 ± 0.02  **0.97 ± 0.03**  0.88 ± 0.02 | **0.20**  **0.40**  0.60  0.80  1.00 | **0.68 ± 0.01**  **0.65 ± 0.01**  0.65 ± 0.01  0.63 ± 0.01  0.63 ± 0.01 | **<0.001 (@ 0.2nA) <0.001 (@ 0.4nA)** |
| **First AP**  **amplitude**  **(mV)** | 0.10  0.20  0.30  0.40  0.50 | 80.2 ± 2.5  81.3 ± 1.5  83.8 ± 1.6  85.4 ± 2.4  91.5 ± 2.2 | 0.20  0.40  0.60  0.80  1.00 | 85.1 ± 1.5  85.3 ± 0.9  84.7 ± 0.7  84.5 ± 0.7  84.5 ± 0.7 | 0.075 (@ 0.2nA) 0.975 (@ 0.4nA) |
| **First**  **AP AHP**  **minimum**  **voltage**  **(mV)** | 0.10  **0.20**  0.30  **0.40**  0.50 | -48.5 ± 3.2  **-47.5 ± 1.1**  -47.2 ± 0.7  **-47.3 ± 0.7**  -48.0 ± 0.2 | **0.20**  **0.40**  0.60  0.80  1.00 | **-66.7 ± 0.8**  **-63.5 ± 0.7**  -61.8 ± 0.7  -60.0 ± 0.8  -59.7 ± 1.0 | **<0.001 (@ 0.2nA) <0.001 (@ 0.4nA)** |
| **First AP**  **AHP**  **depth**  **(mV)** | 0.10  **0.20**  0.30  **0.40**  0.50 | 22.3 ± 0.7  **22.2 ± 0.5**  21.1 ± 0.5  **20.0 ± 0.7**  18.6 ± 1.0 | **0.20**  **0.40**  0.60  0.80  1.00 | **2.8 ± 0.8**  **5.8 ± 0.8**  8.0 ± 0.8  8.2 ± 0.7  8.3 ± 0.8 | **<0.001 (@ 0.2nA) <0.001 (@ 0.4nA)** |
| **First AP**  **Threshold**  **(mV)** | 0.10  **0.20**  0.30  **0.40**  0.50 | -44.6 ± 0.9  **-44.8 ± 0.5**  -45.5 ± 0.6  **-46.1 ± 1.2**  -53.1 ± 0.1 | **0.20**  **0.40**  0.60  0.80  1.00 | **-57.2 ± 0.8**  **-55.1 ± 0.9**  -53.1 ± 1.1  -53.8 ± 1.1  -54.5 ± 1.2 | **<0.001 (@ 0.2nA) <0.001 (@ 0.4nA)** |

**Table S4: Intrinsic neuron excitability**

| **Features name** | **Current**  **(nA)** | **mouse** | **Current**  **(nA)** | **rat** | ***p*** |
| --- | --- | --- | --- | --- | --- |
| **Spike count**  **(n)** | 0.10  **0.20**  0.30  **0.40**  0.50 | 0.7 ± 0.2  **5.1 ± 0.5**  6.9 ± 0.4  **7.9 ± 0.4**  9.2 ± 0.5 | **0.20**  **0.40**  0.60  0.80  1.00 | **1.6 ± 0.3**  **3.8 ± 0.5**  6.6 ±0.6  9.2 ± 0.6  11.4 ± 0.7 | **<0.001 (@ 0.2nA)**  **<0.001 (@ 0.4nA)** |
| **Mean**  **frequency**  **(Hz)** | 0.10  **0.20**  0.30  **0.40**  0.50 | 3.9 ± 1.3  **27.5 ± 2.5**  36.7 ± 2.1  **42.3 ± 2.0**  49.3 ± 2.6 | **0.20**  **0.40**  0.60  0.80  1.00 | **3.9 ± 0.7**  **9.6 ± 1.2**  16.5 ±1.4  23.0 ± 1.5  28.4 ± 1.7 | <0.001 (@ 0.2nA)  <0.001 (@ 0.4nA) |
| **Adaptation index** | 0.10  **0.20**  0.30  **0.40**  0.50 | 0.38 ± 0.19  **0.10 ± 0.01**  0.08 ± 0.01  **0.06 ± 0.01**  0.04 ± 0.01 | **0.20**  **0.40**  0.60  0.80  1.00 | **0.23 ± 0.02**  **0.16 ± 0.02**  0.19 ± 0.02  0.14 ± 0.01  0.11 ± 0.01 | <0.001 (@ 0.2nA)  <0.001 (@ 0.4nA) |


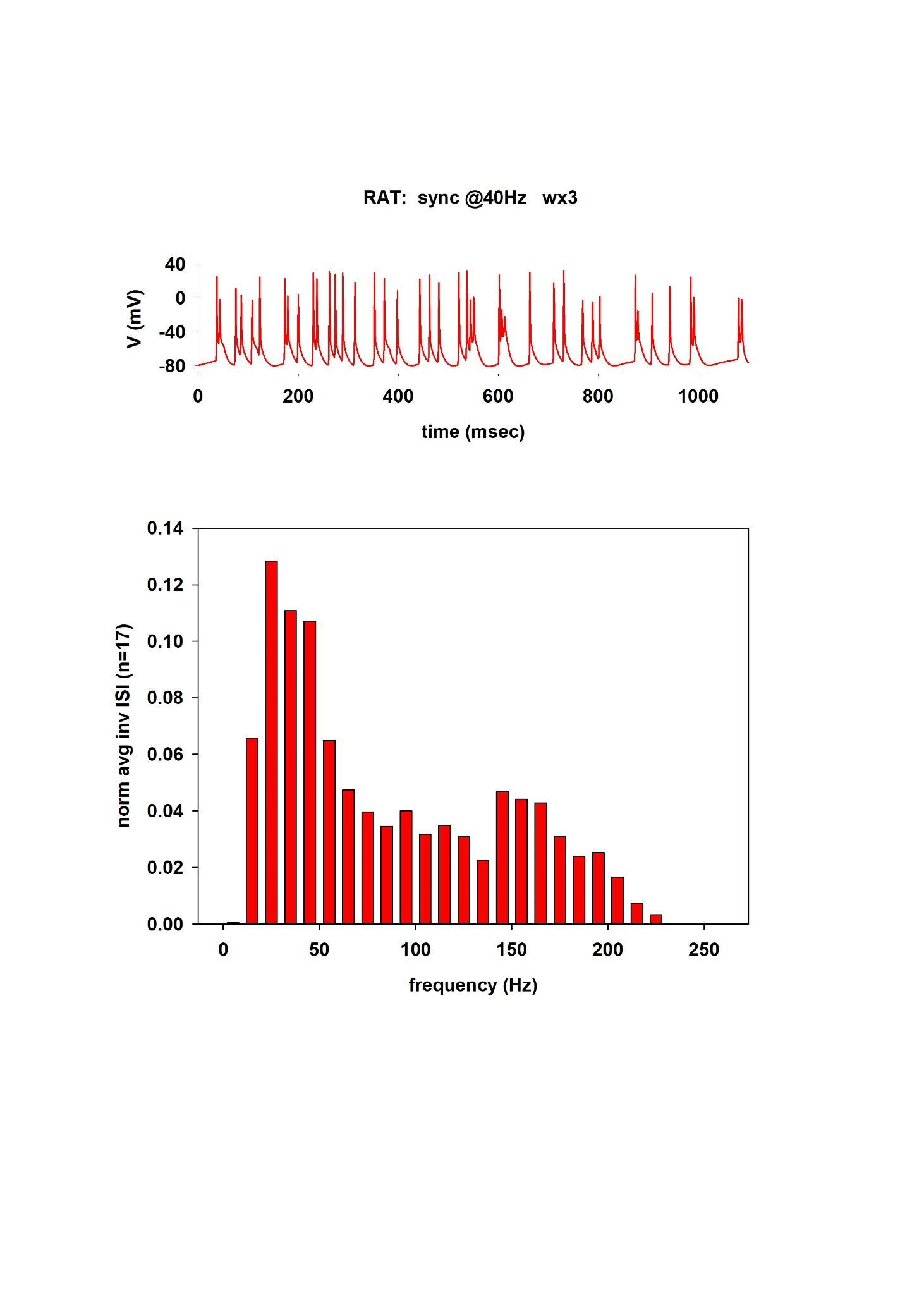


**Fig. S1.** *(top) Firing activity of rat computational model oh140807_A0_idG for 40Hz synchronous activation with synaptic weight of 0.075 nS; and (bottom) corresponding distributions of the ISI ^-1^ as a function of the average activation frequency.*

**
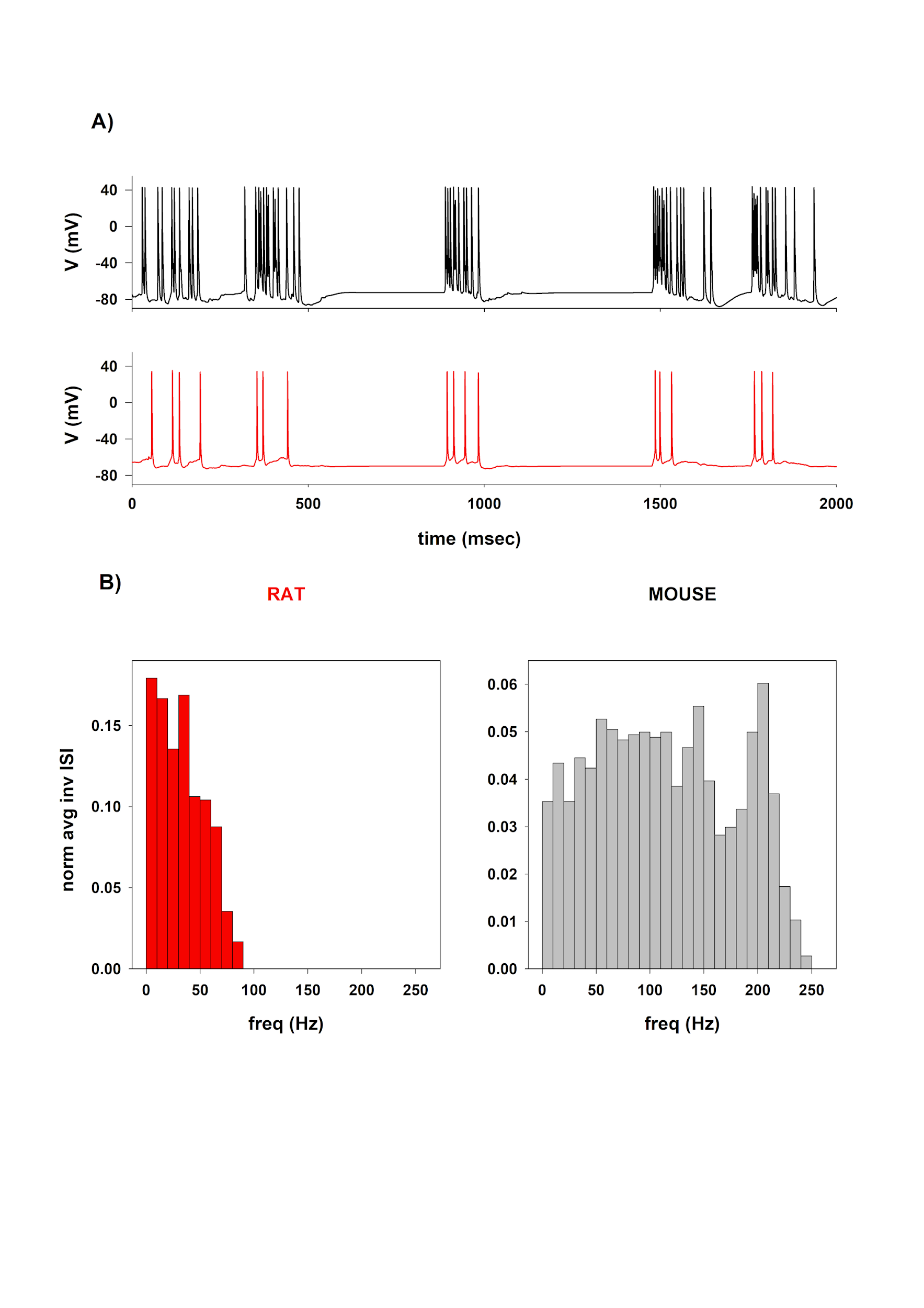
**

**Fig. S2. *A)*** *Comparison between the firing activity charactering the hippocampal CA1 computational models of mouse (cell 150217_A3_idB, black traces) and rat (cell oh140807_A0_idG, red traces) for asynchronous burst stimuli (10 pulses with intra-burst frequency at 80 Hz) with low inter-burst frequency (4Hz). Simulated distributions of the instantaneous AP firing frequency (calculated as the reciprocal of the Inter-Spike Intervals, ISI ^-1^) as a function of the average activation frequency of* ***B)*** *asynchronous bursting synaptic inputs for the rat and mouse.*
